# Incorporation of complementary and traditional medicine in ICD-11

**DOI:** 10.1186/s12911-022-01913-7

**Published:** 2022-06-30

**Authors:** Bill Reddy, Arthur Yin Fan

**Affiliations:** 1 Integrative Health Policy Consortium, 712 H Street NE Suite 1108, Washington, DC 20002 USA; 2 McLean Center for Complementary and Alternative Medicine, Virginia Institute of Integrative Medicine, Vienna, VA USA

**Keywords:** Traditional Chinese medicine, Acupuncture, Complementary medicine, Traditional medicine, ICD-11, International classification of diseases

## Abstract

Traditional medicine (TM) is practiced in various forms in over 180 countries. Despite this, health information systems on TM are limited. Consistent with this, the World Health Organization’s (WHO) international classification for diseases (ICD) has not to date included TM concepts. This is now changing, as the WHO has endorsed the reflection of TM paradigms in the new 11th Revision of ICD (ICD-11). Although some countries have had national Traditional Medicine classification systems for many years, information from such systems has not been standardized nor been made available globally. By including TM within the ICD, international standardization will be possible allowing for measuring, counting, comparing, formulating questions and monitoring over time. ICD-11 is a classification system for the twenty-first century, and it now provides an opportunity for interested users to integrate the coding of diagnostic concepts from both TM and Western Medicine. This paper describes the new TM classification in ICD and demonstrates through coding examples how to code TM concepts alongside Western Medicine concepts.

## Background

Integrative health, as defined by the Integrative Health Policy Consortium, a U.S.-based 501c(4) not for profit, is *a collaborative, comprehensive, person-centered approach to health creation and disease care that addresses all factors impacting health, including social determinants, and embraces all evidence-informed disciplines, both conventional and complementary, in order to achieve optimal health and well-being* [1]. The World Health Organization (WHO) defines Traditional Medicine (TM) as *the sum total of the knowledge, skill, and practices based on the theories, beliefs, and experiences indigenous to different cultures, whether explicable or not, used in the maintenance of health as well as in the prevention, diagnosis, improvement or treatment of physical and mental illness* [2]. Based on the definitions above, the future of health service delivery is the efficient blend of both traditional and conventional medicine where patient and practitioner work together toward their shared vision of health promotion and disease prevention in accordance with the WHO traditional medicine strategy [3]. The WHO strategy also encourages member states to integrate traditional medicine practices into their healthcare systems in addition to regulations and research. The current ICD-10 coding does not contain any traditional medicine diagnoses. The new ICD-11 code set will be inclusive of these, recognizing the value of Traditional Medicine in health promotion and disease management.

Traditional medicine of one form or another is used by 82% of the world’s population [4]. Chinese medicine is practiced in more than 180 countries, and 93% of the member states of the United Nations have acupuncture practice with 18 offering insurance coverage of acupuncture and moxibustion [5]. There are approximately more than 1000 Chinese medicine educational institutions, more than 100,000 clinics, and more than half a million practitioners worldwide [3, 5].

Acupuncture and Traditional Chinese Medicine (TCM) is growing by leaps and bounds in the US. Prompted by the ongoing opioid crisis, acupuncture has been referenced in U.S. legislation, guidelines and policy documents in the past several years. The U.S. National Governors Association released a document entitled, “Expanding Access to Non-Opioid Management of Chronic Pain: Considerations for Governors” in 2020 endorsing acupuncture, spinal manipulation, massage, cognitive behavioral therapy and yoga [6]. In due course, both Medicare and Medicaid will cover a variety of non-pharmacologic management approaches to chronic and acute pain. Eventually, they will expand acupuncture coverage beyond musculoskeletal pain with the primary considerations being cost effectiveness, safety, and health outcomes. Since January, 2020, Medicare is covering acupuncture for chronic low back pain. ICD-11 can offer the framework to capture the length and breadth of conditions treated in the U.S. and elsewhere.

Since more than 90% of the 38,000 U.S. acupuncturists practice privately, and the majority being cash practices [7] (similar to EU practitioners), many acute and chronic health conditions go undocumented, and there is little data relating to patient safety, so that complications and interactions of TM with western medicine can be monitored. In addition, the inclusion of Chinese medicine diagnoses may enable the comparison of diagnostic, clinical outcome, adverse event, and epidemiological information across medical systems as well as enabling unification of the conventional and traditional medicine classifications for diagnosis and interventions. Unfortunately, there is no “one to one” correspondence from TM medical diagnoses to western medical diagnoses. One western medical diagnosis such as essential hypertension has a number of corresponding TM differential diagnoses, and one TM pattern such as Liver Yin deficiency corresponds to a variety of western medical health problems. One of the duties of the World Health Organization is to document mortality and morbidity, and they should include the millions of people worldwide seeing TM practitioners. ICD-11 information can also inform the development of equitable health policy. The United Nations Permanent Forum on Indigenous Peoples wrote in a 2011 document that, *“indigenous peoples are essentially invisible in the data collection of many international agencies and in most national censuses, the disparities in their health situation as compared to other groups continue to be obscured.”* [8] Dr. Houston, a leader in TM stated that, *“If you do not count traditional medicine, then traditional medicine does not count.”*

## ICD-11 chapter 26

Taking into account the above discussion, the WHO has chosen to reflect Traditional Medicine paradigms in the new International Classification of Diseases 11th Revision (ICD-11). This has been achieved through the creation of a new chapter, 26, for “Traditional Medicine conditions.” With regard to the frame of reference of the Chapter 26 ICD-11 code set, Traditional Medicine represents the diagnostic language of Chinese Medicine as it is practiced in China, Japan and Korea (ICTM-CJK). Subject matter experts volunteered from the U.S., Australia and Vietnam to help develop terminology standards. The ICTM includes symptoms, signs, disease names and patterns, indications for treatment, and interventions within the chosen Traditional Medicine systems. In the future, there is potential for traditional medicine content in ICD-11 to encompass other traditional medical systems such as naturopathic medicine and Indian ayurveda.

In 2004, the WHO Regional Office for the Western Pacific began an effort to standardize the 361 acupuncture point locations, terminology and diagnosis that led to the development of the first international standard terminology (IST) for Traditional Medicine [9]. A total of 3259 technical terms were compiled from Traditional Chinese Medicine (TCM), Japanese (Kampo), Korean (KM) and Traditional Vietnamese medicine (TVM) as well as 153 references of traditional/classical medical texts published in those countries [10]. These efforts, along with the development of the International Classification of Traditional Medicine (ICTM), formed the foundation of the development of Chapter 26 in ICD-11.

Although some countries have had national Traditional Medicine classification systems for many years, information from such systems has not been standardized or available globally. By including Traditional Medicine within the ICD, international standardization will be possible allowing for measuring, counting, comparing, formulating questions and monitoring over time. Chapter 26, Supplemental Chapter Traditional Medicine Conditions – Module 1 contains several sections. It begins with a short description, presents some symptomatology definitions, TM aetiology and pathogenesis. From there it lists 150 TM disorders and 196 patterns (Table 1). In the reference guide, practitioners with interest in coding TM concepts are required to first include an ICD-11 diagnosis code (from Chapters 01–25) that can then be combined with a TM1 pattern/disorder from Chapter 26.

**Table 1:** Structure of traditional medicine coding

Traditional medicine	
Patterns	Disorders
Principle-based patterns (TM1) *(SE70-SE7Z)* Environmental factor patterns (TM1) *(SE80-SE8Z)* Body constituents patterns (TM1) *(SE90-SF4Z)* Organ system patterns (TM1) *(SF50-SG1Z)* Meridian and collateral patterns (TM1) *(SG20-SG5Z)* Six stage patterns (TM1) (SG60-SG6Z) Triple energizer stage patterns (TM1) *(SG70-SG7Z)* Four phase patterns (TM1) *(SG80-SH3Z)* Four constitution medicine patterns (TM1) *(SH40-SH9Z)* Other specified traditional medicine patterns (TM1) *(SJ1Y)* Traditional medicine patterns (TM1), unspecified *(SJ1Z)*	Organ system disorders (TM1) *(SA00-SB2Z)* Other body system disorders (TM1) *(SB30-SD6Z)* Qi, blood and fluid disorders (TM1) (SD70-SD7Z) Mental and emotional disorders (TM1) *(SD80-SD8Z)* External contraction disorders (TM1) *(SD90-SE2Z)* Childhood and adolescence associated disorders (TM1) *(SE30-SE3Z)* Other specified traditional medicine disorders (TM1) *(SE5Y)* Traditional medicine disorders (TM1), unspecified *(SE5Z)*

Traditional Chinese Medicine uses a conceptual framework that formulates signs and symptoms into patterns. The four diagnostic methods commonly used in an ancient and modern patient intake examination include observation (or inspection), auscultation and olfaction, questioning, and palpation (which may include abdominal, meridian and various pulses). Diagnoses can fall under the broad categories of Zang Fu organ system (Heart, Liver, Spleen, Lung, Kidney, Gallbladder, Stomach, Small Intestine, Large Intestine, Bladder and San Jiao/Triple energizer), environmental factors (wind, heat, cold, damp, dryness, summer-heat), 5 elements (wood, fire, earth, metal and water), constitution, 6 stages (jue yin, shao yin, tai yin, shao yang, yang ming and tai yang), 8 principles (Yin/Yang, hot/cold, interior/exterior, excess/deficiency), 4 levels or phases (wei, qi, ying, xue), and meridian and collateral patterns (Table 2) [11].

**Table 2:** Overview of chapter 26 organization: supplementary chapter traditional medicine conditions – module I

Disorders/patterns	Types of disorders/patterns
Traditional medicine disorders	Organ system disorders
	Liver system disorders
	Heart system disorders
	Palpitation disorders
	Chest Impediment disorders
	Spleen system disorders
	Lung system disorders
	Cough disorders
	Kidney system disorders
	Strangury disorders
	Other body system disorders
	Skin and mucosa system disorders
	Furuncle disorders
	Abscess disorders
	Female reproductive system disorders (including childbirth)
	Menstruation associated disorders
	Pregnancy associated disorders
	Puerperium associated disorders
	Other female reproductive system associated disorders
	Bone, joint and muscle system disorders
	Joint impediment disorders
	Eye, ear, nose and throat system disorders
	Deafness disorders
	Brain system disorders
	Headache disorders
	Wind stroke disorders
	Qi, blood and fluid disorders
	Mental and emotional disorders
	External contraction disorders
	Warmth disorders
	Childhood and adolescence associated disorders
Traditional medicine patterns	Principle-based patterns
	Environmental factor patterns
	Body constituents patterns
	Qi patterns
	Blood patterns
	Fluid patterns
	Essence patterns
	Organ system patterns (TM1)
	Liver system patterns (TM1)
	Heart system patterns
	Spleen system patterns
	Lung system patterns
	Kidney system patterns
	Meridian and collateral patterns
	Main Meridian Patterns
	Extra Meridian Patterns
	Six stage patterns
	Triple energizer stage patterns
	Four phase patterns
	Defense phase patterns
	Qi phase patterns
	Nutrient phase patterns
	Blood phase patterns
	Four constitution medicine patterns
	Large yang type patterns
	Small yang type patterns
	Large yin type patterns
	Small yin type patterns

Within these descriptive patterns are specific disorders, broken down into those related to organ systems, Qi, Blood and fluid, mental/emotional, and exogenous pathogenic factors.

The intention of TM coding is to report at a regional, national and international level on the treatment of TM disorders, collecting data on the use of TM services in addition to the resources utilized. On the research front, ICD-11 codes can reflect the safety and efficacy of TM interventions and support clinical research of the integration of TM with WM. ICD-11 codes will also be used for insurance reimbursement purposes (with or without WM diagnoses). The current coding under healthcare related causes of harm includes PL01: Complementary or traditional medicines associated with injury or harm in therapeutic use, and PK81.3: Acupuncture or related therapies associated with injury or harm in therapeutic use. This can be very valuable to document any adverse events. Hospital systems, assisted living communities, and other institutions are required to document adverse events in contrast to private practices that are generally held to a lower standard, and don’t have standard protocols in place if such an event were to occur. Codes such as PK81.3 and PL01 can offer a path for reporting in the future.

## Examples of traditional medicine coding integrated with diagnostic concepts from western medicine

Table 3 presents a juxtaposition of TM concepts alongside corresponding Western Medicine (WM) diagnoses. The concepts in Table 3 demonstrate that in certain circumstances, TM concepts have a conceptual relationship to WM beliefs (e.g. Wheezing disorder has conceptual overlap with WM concept Asthma). In other instances, the TM concepts will seem rather disparate to WM providers (e.g. Kidney Yang Deficiency Pattern having direct correspondence to knee Osteoarthritis in the WM model).

**Table 3:** Examples of traditional medicine coding integrated with diagnostic concepts from western medicine

TCM diagnosis (from chapter 26)	Western medical diagnosis (from chapters 1–25)
SA81 Wheezing disorder (TM1)	CA23.32 Asthma
SF9G Bladder dampness heat pattern (TM1)	GC08 Urinary tract infection, site not specified
SF97 Kidney yang deficiency pattern (TM1)	FA01 Osteoarthritis of knee
SF70 Spleen Qi deficiency pattern (TM1)	DA22 Gastro-oesophageal reflux disease
SE90 Qi deficiency pattern (TM1)	BD10 Congestive heart failure
SF86 Turbid phlegm accumulation in the lung pattern (TM1)	CA23.32 Asthma
SF6H Heart and gallbladder Qi deficiency pattern (TM1)	7AOZ Insomnia disorders, unspecified
SF93 Kidney Yin deficiency pattern (TM1)	MG30.02 chronic low back pain
SF54 Liver blood deficiency pattern (TM1)	MB48.0 Vertigo
SF61 Heart blood deficiency pattern (TM1)	3A00 Iron deficiency anemia
SF56 Liver wind stirring the interior pattern (TM1)	8B20 Stroke
SF57 Liver Qi stagnation (TM1)	GA34.3 Dysmenorrhea
SF90 Kidney Qi deficiency (TM1)	GA31 Female infertility

The ICD-11 Reference Guide recommends the combined use of TM codes alongside customary diagnosis code concepts that are found in Chapters 1–25 in ICD-11. Importantly, however, we expect that it is TM practitioners who would most often be the ones performing such integrated coding. Conversely, we expect that WM providers would typically not use TM codes, nor operate with TM concepts in their clinical practices.

**Fig. 1:**
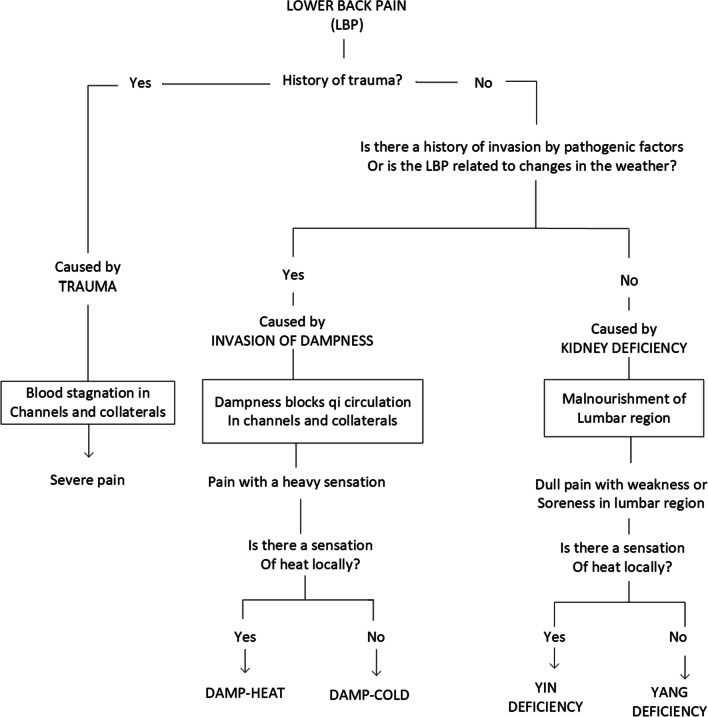
Low back pain (Adapted from: Li Xuemei, Zhao Jingyi. Acupuncture patterns and practice. Seattle: Eastland Press, 1993.)

Figure 1 presents the example of an algorithm, based in TM, for differentially diagnosing low back pain. There are similar diagnosis algorithms common in WM, but demonstrate different diagnostic concepts.

## Controversy surrounding traditional medicine content in ICD-11

**Fig. 2:**
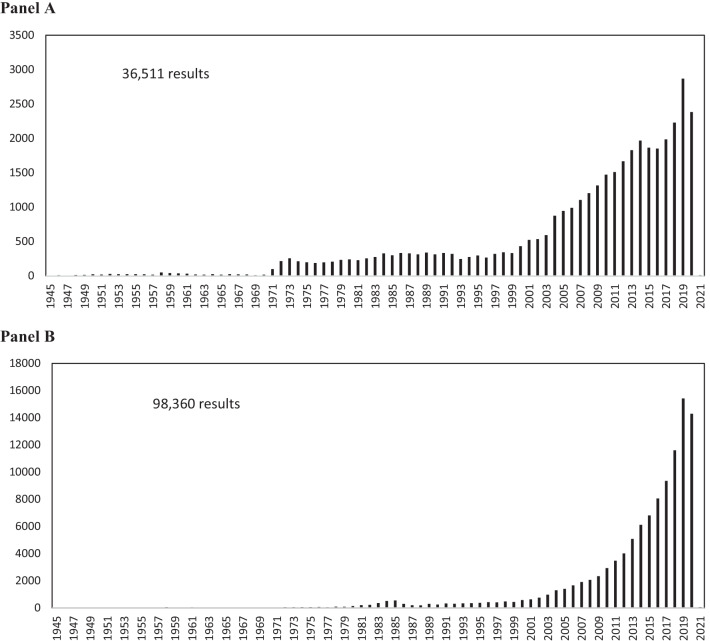
Graph of number of NIH PubMed articles containing the key word “Acupuncture” (**Panel** **A**) and the key word “Traditional Chinese Medicine” (**Panel** **B**)

With the growth of TM use in Western countries, data collection on diagnostic, clinical outcome, adverse events, and epidemiological information across medical systems is likely warranted. Conventional and complementary healthcare providers have varying views regarding the implications of TM coding in ICD-11. In a 2018 Nature article entitled, *Why Chinese Medicine is heading for clinics around the world *[12], Dr. Ryan Abbott said, “Inclusion in ICD-11 is “*a powerful tool for [health-care] providers to say this is legitimate medicine*.” In the same article, Dr. Richard Peto, an epidemiologist and statistician at the University of Oxford, UK stated, “*I thought the WHO was committed to evidence-based medicine *[12].” These differing perspectives on Traditional Medicine paradigms are difficult to reconcile. The WHO decision to incorporate TM concepts in ICD-11 is clearly one that acknowledges a diversity of medical paradigms embraced by the world’s populace. It is not merely about “evidence” per se, but about acknowledging the existence of diverse paradigms surrounding health. It is noteworthy that acupuncture is a well-studied form of TM with more than 36,000 results in the National Institutes of Health (NIH) PubMed using the search term “acupuncture” (Fig. 2, Panel A). Similarly, there are more than 98,000 PubMed results using the term “Traditional Chinese Medicine” with growing numbers in recent years (Fig. 2, Panel B).

In 2019, an editorial in the journal *Nature* suggested that the WHO’s inclusion of TM codes in ICD-11 may backfire [13]. The article starts out by suggesting that traditional Chinese medicine is based on “unsubstantiated theories of meridians and qi.” That statement questions a medical paradigm embraced by a substantial portion of the world’s population, and it flies in the face of many initiatives and agencies that seek to reconcile diverse schools of thought relating to health and disease. Dr. Langevin, Director of the National Center for Complementary and Integrative Health (NCCIH) at NIH is one of the leaders in the field of connective tissue/fascia, mechanical and cellular effects of acupuncture, and meridian pathway research, and she demonstrated that acupuncture meridians are real and measurable [14, 15]. Qi (pronounced chee) has also been maligned by western medical practitioners as being some ancient magical or metaphysical Chinese concept with no research to support its existence, however, the recent science of biophotonics sheds light onto the subject [16, 17]. Another relevant example of bridging thought is Dr. Leonard Wisneski’s textbook entitled “The Scientific Basis of Integrative Health.” It covers in detail the physiologic mechanisms of acupuncture including gate theory, production of endogenous opioids, mechanotransduction, microtrauma, etc. and is required reading in the Academy of Integrative Health & Medicine’s Fellowship program [18]. The notion of integrative health is indeed endorsed by notable agencies such as the NIH, FDA, CMS and HHS in the U.S. The WHO decision to incorporate TM concepts in ICD-11 is seemingly yet another such endorsement of the need to integrate paradigms of thought around health and well-being in the global healthcare arena.

## Conclusion

ICD-11 is a classification system for the twenty-first century. We live in an era where care is increasingly patient-centred and the patient voice is being acknowledged and respected more than it has previously. In the spirit of pluralism and respect for equity, diversity and inclusivity, there is an impetus for the explicit recognition of TM paradigms in health information systems. ICD-11 now provides an opportunity for interested users to integrate the coding of diagnostic concepts from both TM and WM, as demonstrated in the examples presented here.

## Data Availability

Data sharing is not applicable to this article as no datasets were generated or analysed during the current study.

## Abbreviations

CMS: Centers for medicare and medicaid services
FDA: Food and drug administration
HHS: U.S. Department of Health and human services
ICD: International classification of diseases
ICD-10: International classification of diseases, 10th revision
ICD-11: International classification of diseases, 11th revision
IST: International standard terminology
ICTM: International classification of traditional medicine
ICTM-CJK: International classification of traditional medicine, China, Japan & Korea version
KM: Korean medicine
NCCIH: National center for complementary and integrative health
NIH: National institutes of health
TCM: Traditional chinese medicine
TM: Traditional medicine
TVM: Traditional Vietnamese medicine
WHO: World Health Organization
WM: Western medicine
